# Philadelphia County Medical Society

**Published:** 1890-11

**Authors:** 


### PHILADELPHIA COUNTY MEDICAL SOCIETY.

Stated meeting, September 10, 1890.

The Vice-President, John B. Roberts, M. D., in the chair.

Dr. Benjamin T. Shimwell read a paper on

### A NEW METHOD OF DELIVERING THE FETAL HEAD.

Nature's manner of delivering the fetal head has been followed by obstetricians from time immemorial, recognizing the fact that the occiput is born under the symphysis pubis in normal labor. The face and chin stretch the perineal body, then force their way out, requiring an especial amount of care to prevent tearing of this tissue. The extent of injury to the pelvic floor is not properly appreciated; if the superficial tissue of the perineum is safe, the attendant congratulates himself on his possible skill; or, if aware of deeper injury, feels grateful that no apparent injury is shown to the watchful eyes of the nurse or patient's friends.

Thus do thousands get out of the confinement-bed ruined in health; carrying into the future injuries that must of necessity bring ill results. Various plans have been suggested to support and accommodate the perineal body to the oncoming head.

It is strange how often the anatomical construction of the perineum is overlooked, and considered merely as a space-filler. It is by this that so much injury is done. The gynecologist's specialty lives by these results.

These are the reasons, hastened probably by experience gained in the above manner, that has induced me to write this paper. The theory that will be advanced, backed by my application of it in a great number of cases, is evidently new; if not, it has not come to my knowledge by reading or otherwise. The advantage of this method is the saving of the pelvic floor from injury, either superficial or deep. No attempt is made to show expedition, but a modification of the ordinary method of labor changing the direction of the impinging force.

Naegele says that seventy out of every one hundred vertex presentations are in the first position, the other thirty are occiput to right and posterior. The remaining positions are exceedingly rare.

When the head presents in the first position, the body of the child must not be overlooked. The back of the child must present to the front and left, the chest to the back and right; therefore, at right angles with the vertex presentation of the head at the superior strait. The important point in this theory is the rotation of the head to the symphysis pubis. The manner of rotation of the head is mooted. Pajot claims that the shoulders participate in the rotation, but contradicts himself when he further says: "That it is above all the shape of the child's head which decides the character of the movement;" also, "That the occiput will, therefore, be carried forward less on account of the direction of the forces which impel it, than because of the necessity for accommodation of the cephalic surfaces to the pelvic surfaces." All writers admit that after expulsion of the head occurs restitution takes place; that in a case of first position, after the head is delivered, the head turns with its occiput to the left thigh — that is, in the direction that the head presents at the superior strait; this is an untwisting of the neck.

Gerdy claims that this is "an external expression of a movement of the shoulders within the pelvis, by which the biacromial diameter passes from the transverse to the antero-posterior diameter, the head following the internal rotation." The folly of this assertion is on its surface. The head is free and the neck and body are constricted by the vagina and uterus, and if rotation does take place, can we overlook the anatomical relation and action of the vertebræ? Would not the weight of the head allow of the rotation internal without its external manifestations?

Penrose (Hirst, Vol. I., p. 571) says: "While the head has rotated, the body of the child, still in the cavity of the uterus, has been tightly grasped by the firmly contracting walls of the uterus, and has not participated in the movements of the head; hence the shoulders are still oblique at the superior strait, consequently the neck of the child is twisted." The latter theory, according to my experience, is the true one. The fact of the anatomical construction of the cervical vertebræ of the child cannot be overlooked. This arrangement allows of a rotation of one-fourth of its circumference to take place without injury to the spinal cord. Therefore, if Pajot's theory of the accommodation of the cephalic surfaces to the pelvic surfaces, rather than the application of the force, is true, then it can be seen that rotation of the head is possible without the shoulders. Then, again, the head is not free to wobble around the pelvis when it has reached such a condition of flexion; neither is the neck a rigid body depending on the shoulders for its position. If the theory of shoulder rotation is so, then nature's method is superfluous; for, why should the biacromial diameter be changed from its oblique position, which is nearer the antero-posterior diameter, to the transverse, then rotate back again beyond its former position to the antero-posterior.

Playfair believes in partial rotation.

Believing, then, that the shoulders still maintain their oblique position through all the stages of the delivery of the head, what occurs when rotation brings the occiput directly antero-posterior? This has been accomplished by the rotation mentioned of the atlas on the axis vertebra to one-fourth of its circumference; this having occurred, the delivery of the head takes place, then immediately external rotation or restitution occurs, that is, the neck untwists.

The outlet of the female pelvis is four inches antero-posterior and transverse. The antero-posterior is possibly increased a half inch by extension of the coccyx. These measurements are decreased by the soft tissues; this is more marked in the antero-posterior by the rectum and perineal body. As the head in the last act of delivery begins to extend, we have presenting the cervico-frontal diameter, which is four inches; this has to pass through a space that is but four inches, possibly four and a half inches, lessened by the perineal body, which is at this stage excessively stretched and attenuated. As the safety of the perineum is an exceedingly important matter, it occured to me that this might be accomplished by lessening the size of the impinging body and transferring the extending head into another direction. It is the nose and chin that rupture the pelvic floor; therefore, if the direction of this force can be changed to some other point than the junction of the levator ani muscle, it can be easily seen how injury to this muscle can be prevented.

When the labor has reached this stage, I place the woman across the bed on her back, knees well drawn up, then compel her to breathe with the mouth wide open to prevent bearing down. As the head presents in the oblique direction and to reach the antero-posterior diameter it rotates, twisting the neck, the first step in the method is to reëstablish the direction of the first impingement; this is not done until the cervico-frontal diameter is reached; *this must be complete*, then forcing the head into extreme flexion by grasping the presenting occiput by the hand (in non-instrumental labors), I begin my rotation; the first step is to untwist the neck; this accomplished, the head presents cervico-frontal to the left anterior. I then take advantage of the same anatomical construction of the cervical vertebræ that allows of the normal rotation, and rotate one-fourth in the opposite direction, that is, to the left. The cervico-frontal is then transverse, the neck lying on the labia of the left side, the forehead beginning to engage the soft tissues of the right labia. What is now presenting to the antero-posterior diameter, or, what is more important, to the perineum? The biparietal diameter, which measures three and one-half inches, therefore less tension on the perineum. The possibility of delivering the head in the transverse diameter has been questioned. The articulation of the head to the spinal column is wisely arranged; if no other object than birth was intended, it has well served its purpose. The diameters of the extending face are those of a right-angle triangle, the hypothenuse of which is four inches, the perpendicular three inches, the base two and four-fifths inches. The mechanical advantage of this is apparent. If the measurements had been those of a triangle, the impossibility of delivery is easily seen, the head could not be born as long as the perineum existed. The sweep of the extending head would be the same at the chin as at the forehead, and the perineum would be torn in every case and in every succeeding labor; but the measurements are those of a right-angle triangle, and of a necessity the chin must recede when complete extension takes place; so when extension is made in the transverse diameter of the inferior strait, the chin does not impinge on the ramus of the ischium.

Having got the head into this position, I begin the last stage of the delivery of the head. The head has been all this time in extreme flexion, then extension is performed, the soft tissues of the labia push aside, and nose follows on forehead, chin on nose; delivery is complete, and the pelvic floor is safe. The head then untwists to its normal position.

DISCUSSION:

Dr. E. E. MONTGOMERY: I think that the members of the Society are greatly indebted to Dr. Shimwell for the graphic presentation of this method of dealing with the delivery of the head and effort to save the perineum. This certainly is a violation of that old principle which has been handed down the ages that "meddlesome midwifery is bad." When we consider that all progress in obstetrics and every step in advancement has been in violation of this principle, this thought may not be considered an objection to this procedure, which certainly seems to be one which should be serviceable. But, not having had experience myself, I am unable to say more than these few words in commendation of it.

Dr. J. M. BALDY.—It seems to me that the remarks of Dr. Montgomery, in regard to meddlesome midwifery, are true as regards pathological processes, but not as regards physiological processes. Certain it is that in almost everything which we have attempted to interfere with physiological processes, we have found that they have been carried on a great deal better by nature herself than by any so-called improvement that we have made on her. If nature had meant that the head should be delivered in the transverse diameter of the outlet, she would have given us some indication of such desire. On the contrary, she has shown us very clearly and distinctly that the head was to be delivered in the antero-posterior diameter. It is probable that the head can be delivered in the transverse diameter, as Dr. Shimwell has pointed out, if all the measurements are of average size; but all of us know perfectly well that it is the exceptional head that we come across, and not the typical head. Many of the heads are large, and the higher we get in the stage of civilization the larger the head. The normal head may go through, but I doubt not that Dr. Shimwell will run across many cases that he will not be able to deliver in the transverse diameter. Unless the head will pass easily, we have here no room for extension of the outlet. It is a fixed quantity bound by bony walls— the ramus of the ischium on both sides — and there can be no distention. On the contrary, in the natural methods of delivery we have free room for extension taking place through the perineal body and the soft part of the lower part of the pelvis. Now, it may be that there is danger to the levator ani muscles from over-distention, but at the same time I conceive, and it has been my experience, that the danger to these muscles is greater in proportion to the amount of interference we give to the perineum. In other words, we have here a hard body starting from a given point and progressing at a certain angle to a certain point at which it meets a plane of resistance, that plane of resistance being the soft parts of the pelvic floor, and, if you will, principally the levator ani muscles. There is a well-known physical law, that any body moving in a given direction and meeting with an obstacle, will be deflected at a certain angle. We have this occurring in delivery of the head. The head comes down and meets a resistance, which, although not a fixed resistance, is sufficient to cause deflection in the line of least resistance. This line of least resistance is the opening of the vulva. If resistance is given to the head at that point, the head is prevented from bulging through the vulvar orifice, and, the *vis a tergo* being still active, must be spent at some place, and that place is at the point of contact of the head with the pelvic floor. Taking the head, which is bulging the perineum and presenting at the vulva, we hold it back by pressure on the perineum, or by some other method; then we are going to have the greater part of the *vis a tergo* exerted at this one point. These soft tissues of the pelvic floor are capable of yielding to a certain point, and, when they come to that point, they are going to give, and there will be a tear of the levator ani muscles and the other tissues involved. This is where, I believe, the vast majority of tears of the perineum come in. All instructors teach that the head should be held back in some way or other, so that the vulvar orifice is not allowed to expand and the head protrude, as nature intended; and by this misapplied force we bring about the accident we are trying to avoid. I have found in the cases in which I allowed nature to take her course almost entirely, keeping the fingers from the head and perineum, excepting to make slight pressure and lift the head up against the pubic arch, that they have done better and I have had fewer tears, and those that have taken place have been of a minor degree as compared with those where I tried to prevent injury by supporting the perineum. Any support of the perineum whatever is pernicious. I believe that all the teachers and all the books are at fault in that respect. Nature did not mean to have the head held back, and have the whole force spent on one part, when we have the elasticity of all the soft parts well anchored, so as to yield and to give room for the head to pass. Supporting the perineum prevents the proper stretching of these tissues, and prevents any good they may do in bulging the perineum and forcing the vulvar orifice open.

Dr. SHIMWELL.—I am exceedingly sorry that some of my friends who have used this method successfully have not spoken. Dr. Baldy has raised the objection that a large head could not be born transversely, but the same objection applies to the antero-posterior position as well. He overlooks the fact, demonstrated by the mathematical figure, that we gain, as the chin is delivered, a fraction over one and a quarter inches. The head is born without impinging on the soft tissues of the pelvis.

I have tried this method successfully for a year and a half, both in primiparæ and in multiparæ. I have used it both in cases terminated without instruments and in those where the forceps have been required on account of loss of tone or from malformation on the part of the head or of the pelvic outlet.

In regard to nature—nature is not always a good worker. If so, why should we have a disproportion between the head and the pelvis? The outlet should be made equal to the head. With regard to the increase in the size of the head with advancing civilization, I know that; but is the pelvis unchanged? Is it not rather lessened? Has it not changed its size and shape?

The points advanced are, I think, no argument against the method. It is a safe method; it is an easy method; and the delivery is accomplished with perfect safety to the child and to the mother.

